# Environmentally induced dynamics between intraindividual variation and trait means across vegetative, phenological, and floral traits

**DOI:** 10.1002/ajb2.70244

**Published:** 2026-08-07

**Authors:** Charlotte Møller, Martí March‐Salas, Mar Sobral, Judith Haase, Pieter De Frenne, J. F. Scheepens

**Affiliations:** ^1^ Plant Evolutionary Ecology, Faculty of Biological Sciences Goethe University Frankfurt Max‐von‐Laue‐Str. 13 Frankfurt am Main 60438 Germany; ^2^ Department of Plant and Environmental Sciences University of Copenhagen Thorvaldsensvej 40 Frederiksberg C 1871 Denmark; ^3^ Area of Biodiversity and Conservation, Department of Biology and Geology, Physics and Inorganic Chemistry University Rey Juan Carlos‐ESCET Tulipán s/n. 28933 Móstoles Madrid Spain; ^4^ Geography Department Universidade de Santiago de Compostela Santiago de Compostela 15782 Spain; ^5^ Forest & Nature Lab, Faculty of Bioscience Engineering Ghent University Geraardsbergsesteenweg 267 Gontrode 9090 Belgium

**Keywords:** clonal plant, forest understory herbs, *Galium odoratum*, intraspecific variation, phenotypic plasticity, plant functional strategies, subindividual variation

## Abstract

**Premise:**

Intraindividual variation in reiterated structures (e.g., leaves) represents an important dimension of phenotypic variation that contributes to functional diversity and the dynamics of ecological communities. It can be expressed differently across plant traits, but how environmental drivers affect intraindividual variation in different trait types remains unknown.

**Methods:**

Here we subjected *Galium odoratum* to drought and shading treatments with a full‐factorial experimental design to investigate environmental induction of intraindividual variation in vegetative, phenological, and floral traits and the dynamic relationships between trait mean responses and their variation.

**Results:**

Intraindividual variation differed in magnitude between trait types, with vegetative traits having the highest variation and floral traits the lowest. Intraindividual variation differed among the environmental treatments with floral traits having the highest variation under control conditions, whereas vegetative traits had the highest amount of variation under the combined drought and early‐shading treatment. Relationships between intraindividual variation and trait means varied across most traits and treatments, indicating environmentally induced dynamics in trait expressions.

**Conclusions:**

Intraindividual variation was environmentally inducible and dynamic depending on the treatment, suggesting that plastic responses in intraindividual variation may be an important component for mechanistic adjustments of plants to environmental stresses.

Trait variation within individuals in reiterated homologous structures (for example, leaves) can be referred to as intraindividual variation (Herrera, 2009). Despite its magnitude and ubiquity, intraindividual variation has been largely overlooked, although recent research has shown the relevance of intraindividual trait variation for functional diversity and ecosystem functioning (Sobral, 2023; Sánchez‐Bermejo et al., 2023, 2024). However, whether and how the intraindividual functional trait space (variation and means of multiple integrated traits) reorganizes in response to environmental variation remains unknown.

Within individual plants, variation in vegetative traits such as leaf size and plant height has been found to be highly plastic and affected by environmental cues (Møller et al., 2023). However, floral traits within individual plants are known to be less variable than vegetative traits (Herrera, 2009; Saiz‐Blanco et al., 2025), possibly because they are under stronger selection (e.g., by pollinators) compared to leaf traits (Paglia et al., 2023). Leaf traits are generally more plastic than floral traits in response to abiotic factors (Brunet and Van Etten, 2019) such as light and water availability or their combination (Navas and Garnier, 2002; Lambrecht et al., 2017). However, intraindividual variation in floral (Paglia et al., 2023) and phenological traits (March‐Salas et al., 2021; Gómez et al., 2023) such as flowering start and flower longevity could allow plants to enhance their fitness and shift their pollination niche in stressful conditions (Ehrlén and Valdés, 2024). Given that all traits are integrated within the organismal phenotype by mechanistic and energetic constraints, the intraindividual functional space likely varies under environmental stress. Yet, the extent to which different traits (e.g., phenological responses, reproductive traits, and leaf traits) vary intraindividually and how variation is environmentally induced is not yet well understood.

Relationships between intraindividual variation and trait means (usually indicating the expression and performance in a given trait) can vary depending on the environment and available resources. A positive relationship can be observed when traits respond in a coordinated way due to shared underlying genetic, developmental, or environmental drivers—also known as phenotypic integration (Pigliucci, 2003; Matesanz et al., 2021)—or simply when resources are not limited. A neutral or absent relationship suggests potential independence of the involved traits or perhaps hidden constraints. And lastly, a negative relationship can indicate a functional constraint or even a trade‐off (Southwood, 1988), in which the improvement of one trait is associated with the reduction or limitation of another. Ultimately, variation in environmental conditions can induce changes in the intraindividual trait functional trait space and in their relationship to average trait values. However, the dynamics between intraindividual variation and their trait averages remain poorly studied.

Here, we explored the expression of intraindividual variation in different trait types and the environmentally induced dynamics between intraindividual trait mean responses and their variation in a multidimensional context. More specifically, we compared intraindividual variation of vegetative, phenological, and floral traits of the herbaceous, forest‐floor plant *Galium odoratum* (Rubiaceae). Forest‐understory herbs are increasingly subject to climate‐change‐driven drought (Dai, 2013; Büntgen et al., 2021), also in temperate forests (Trenberth et al., 2014; Millar and Stephenson, 2015; Dai et al., 2018). Furthermore, long‐term phenological records from North America indicate an altered light regime due to changes in the phenology of tree leaf‐out that has created an approximately 2‐week asynchrony between the trees in the overstory and the herbs in the understory (Heberling et al., 2019). In this context, we tested how experimental drought and the timing of shading, as key global‐change drivers in European forests (De Frenne, 2024), induce shifts in intraindividual variation, within and among various trait types and in the relationship between trait variation and trait means. Thus, we asked (1) whether the amount of intraindividual variation varies among different vegetative, phenological, and floral traits; (2) whether this variation and the corresponding trait mean responses are induced by environmental stressors in the form of treatments aimed to simulate environmental changes in forest environments; (3) and whether and how relationships between intraindividual variation and trait mean responses within and across trait types are altered by environmental stress in a functional trait space.

## MATERIALS AND METHODS

### Study species and experimental system

We used *Galium odoratum* (L.) Scop. (Rubiaceae) as our study species. *Galium odoratum* is a perennial forest forb occurring in Europe, reaching heights of 10–30 cm, and flowering in April–June. Reproduction can occur sexually via seeds, but *G. odoratum* also heavily relies on vegetative spread through stolons (Frederiksen and Rasmussen, 2006). The leaves are lancet‐shaped, widest at the middle or closer to the leaf apex and mostly appearing eight per whorl. The flowers are white, small, funnel‐shaped and occur in cymes (Lim, 2014). The species' primary habitat is shaded woodlands with moist and rich soils (Frederiksen and Rasmussen, 2006).

In May 2020, using the experimental setup of Møller et al. (2023), we sampled five individuals of *G. odoratum* each from 21 populations (100 × 100 m forest plots; 105 plants total) distributed in three large‐scale research regions of the Biodiversity Exploratories (www.biodiversity-exploratories.de) across Germany: Schwäbische Alb (South Germany), Hainich‐Dün (Central Germany), and Schorfheide‐Chorin (North Germany).

The Schwäbische Alb is underlain by calcareous bedrock and represents the highest elevations among the sites, reaching up to 860 m above sea level (m a.s.l.). It is characterized by mean annual temperatures of 6–7°C and annual precipitation ranging from 700 to 1000 mm. The forests are predominantly small‐scale mosaics and are subject to relatively high management intensity.

The Hainich‐Dün region is likewise dominated by calcareous substrates but occurs at lower elevations, with a maximum of 550 m a.s.l. Mean annual temperature ranges from 6.5 to 8°C, and mean annual precipitation ranges from 500 to 800 mm. Vegetation is primarily composed of extensive beech forests, managed largely under a selection forestry system.

In contrast to the other regions, the Schorfheide‐Chorin region is characterized by sandy soils and low elevation (approximately 140 m a.s.l.), comparatively warmer mean annual temperatures (8–8.5°C) and lower annual precipitation (500–600 mm). The forests are mainly age‐class stands, including old‐growth beech forests and mixed‐species forests (Fischer et al., 2010).

The five *G. odoratum* individuals per population (hereafter referred to as genets) were sampled with a minimum interindividual distance of 10 m to ensure sampling genetically different individuals (Ziegenhagen et al., 2003). Each genet was vegetatively propagated by stolon separation into four ramets and transplanted into multitrays (51.5 cm wide, 33.5 cm long), each with 54 cells (5.0 cm diameter, 5.5 cm depth), filled with potting soil (CL T torffrei, Einheitserde, Sinntal‐Altengronau, Germany) and placed in an outdoor common garden for establishment and growth until November 2020. Subsequently, all ramets were transferred to 1.5‐L pots of potting soil (Typ T, Struktur 1b, Hawita, Vechta, Germany), allowing for natural vegetative ramet production until the end of the experiment. In spring 2021, we relocated all pots to a foil tunnel with open ends to exclude natural precipitation.

### Experimental shade and drought treatments

At the start of the original experiment in 2021, shade and drought treatments were applied in a full‐factorial design: The four ramets from each genet was placed in a different treatment condition: (1) control, (2) drought, (3) early shading, or (4) combined drought and early shading. To simulate 2‐week shaded forest understory environment and climate‐induced mismatch between forest over‐ and understory, we applied shade cloth over the foil tunnel in two layers (45% shading for each layer, resulting in 90% total shading). Within one treatment, the two shade cloth layers were applied 1 week apart. The first layer of the early‐shading treatment was applied ~2 weeks before the anticipated leaf‐out of beech and oak trees in the surrounding area (that is, on 12 April 2021 and 11 April 2022). The control shade cloth was applied when natural leaf‐out of the surrounding trees was observed in the area (Frankfurt am Main, Germany) on 30 April in 2021 and 25 April in 2022 (again, the two layers were applied 1 week apart). The drought treatment was applied to all plants when the first plant started flowering (on 7 May 2021 and 30 May 2022). This drought treatment was applied as a single event in which all watering ceased until 50% of the plants were wilting (which took approximately 3 weeks). Then regular watering (as in the control treatment) resumed for all pots. Control plants regularly received water as needed throughout the experiment (during flowering, they were watered almost daily due to the hot summer). All watering was from overhead irrigation.

### Mean and intraindividual trait measurements

Measurements of vegetative traits in 2021 were published by Møller et al. (2023), so only the results for vegetative, phenological, and floral traits measured in 2022 are presented here. The phenological traits included start of flowering and flower longevity, and the floral traits included flower size and petal size. Flowering start was defined as when the first flower had visible anthers. Plants were then checked every Monday, Wednesday, and Friday between 1 April and 1 June 2022, for the first 15 flowers per ramet. Two days after flowering start had been recorded for an individual flower, the diameter of the flower and length of a petal were measured with calipers to the nearest 0.1 mm for the same 15 flowers. Flower longevity (number of days that a flower was open until the petals wilted) was tracked every Monday, Wednesday, and Friday between 1 April and 6 June 2022. From the start of the experimental setup in 2021, to the time we took our measurements in 2022, 22 of the original 135 ramets included by Møller et al. (2023) had died. had In the end, we measured 1621 flowers across a total of 113 ramets (mean number of flowers measured per ramet ± SD = 14.26 ± 3.83) from among 65 genets (mean number of ramets per genet ± SD = 1.74 ± 0.83) from 21 populations (mean number of genets per population ± SD = 3.10 ± 1.55) (Appendix S1).

The measured vegetative traits included leaf length and leaf width, measured with a caliper to the nearest 0.1 mm on one randomly chosen leaf from the top leaf whorl on a maximum of five ramets per pot (mean number of ramets measured per pot ± SD = 4.94 ± 0.28) after natural vegetative reproduction had occurred within each pot). We only measured leaves from the top whorls of the shoots because they are closest to the flowers, controlling for effects of plant development and spatiotemporal variation in the microenvironment when comparing floral and leaf traits. Due to the immense workload, vegetative traits were measured after the flowering peak; thus, additional ramets had died after the phenological and floral trait measurements, resulting in a total of 425 leaves measured (one leaf per ramet because we only worked with the top whorl) across a total of 86 ramets (mean number of leaves measured per ramet ± SD = 4.94 ± 0.28) from 52 genets (mean number of ramets per genet ± SD = 1.65 ± 0.76) from 15 populations (mean number of genets per population ± SD = 3.47 + 1.41) (Appendix S2).

Since each of the five genets that we sampled from each population was subsequently divided into four different ramets, then grown in different pots, and with different treatments, we did not classify the intragenet level among pots and across treatments as an intraindividual level. Therefore, as an estimate of intraindividual variation, we either used the coefficient of variation (CV; calculated as the standard deviation divided by the mean of a specific trait at the intraramet level) or the standard deviation as a proxy for intraindividual variation depending on our question.

### Data analyses

R version 2023.09.1 (R Core Team, 2023) was used for all analyses.

#### Differences in intraindividual variation among plant traits

To address our first objective and investigate how the amount of intraindividual variation (here calculated as CV) differed between vegetative, phenological, and floral traits, we combined the CV of all traits and tested for the effects of the specific trait type on the CV using a linear mixed‐effects model (LLM). The CV calculated for each trait within the complete data set (CV_ALL_) was used as the response variable with the trait identity (flower size, petal size, flowering start, flower longevity, leaf width, and leaf length) as the explanatory factor and genet nested within population, nested within region, as the random factor (region/population/genet). Despite some ramet death, we retained a minimum of two ramets per genet, warranting the inclusion of genet as a random effect to uphold the hierarchical experimental design. The LMM was run using the function lmer from the R package lme4 (Bates et al., 2015), and the LMM test results were obtained by applying the function Anova from the R package car (Fox et al., 2007). We used the function emmeans from the R package lsmeans to run a post hoc Tukey test to identify which trait CVs differed significantly from each other (Lenth and Lenth, 2018).

#### Treatment effects on intraindividual variation and trait means

To address our second objective and investigate the effect of environmental stressors, we tested for the effects of the treatments on each specific trait separately. Here, the trait CV was used as the response variable, with trait mean, shade and drought treatments, and the interaction between the shade and drought treatments as fixed factors. Again, genet nested within population, nested within region, was used as a random factor. Normality and homoscedasticity of all model residuals were evaluated visually with qq‐plots and histograms. When at least one of these assumptions was violated, the response variable was square‐root‐transformed. This transformation was applied to flowering start and petal size. Subsequently, predicted values were extracted from the models for visualization and further investigation of significant treatment effects with post hoc Tukey tests using the function emmeans in the R package lsmeans (Lenth and Lenth, 2018). The same analyses were repeated with the trait means as response variables and the corresponding trait CV as a fixed factor.

#### Relationships between intraindividual variation and trait means

For our third objective, to investigate whether and how relationships between intraindividual variation and trait mean responses are environmentally induced, within and across trait types, we ran a principal component analysis (PCA) using the function prcomp to identify the functional trait space of all the measured traits in concert. Here, we use SD instead of CV as a measure of intraindividual variation to account for the typical negative relationship between CV and the mean (Pélabon et al., 2020). The SD is a scale‐dependent, absolute measure, ensuring that the observed relationships will not be confounded by inherent mathematical coupling. We ran the PCA for each treatment independently: control, drought, early shading, and combined drought and early shading (*N* = 26, 33, 28, and 26 individuals, respectively). Each individual trait SD and trait mean was log‐transformed and scaled for appropriate comparison. Furthermore, the PCA loadings, representing the trait SDs and trait means, were extracted for the first two axes for each treatment PCA (control, Appendix S3; drought, Appendix S4; early shading, Appendix S5; combined, Appendix S6). To test for the significance of the PCA loadings, we applied permutation‐based methods using the function PCAtest from the R package PCAtest (Camargo, 2022), which allowed us to evaluate whether the observed loadings significantly exceeded values expected under null models.

Lastly, we computed matrices of pairwise correlations between trait means and trait SDs for each of the four treatment combinations to check the significance, strength, and direction of the relationships between intraindividual trait variability, trait means, and their combination. Pairwise Pearson correlation coefficients were calculated using the function cor to identify any significant relationships for further interpretation. The correlation structures were visualized using heat maps.

## RESULTS

### Differences in intraindividual variation among plant traits

**Figure 1:**
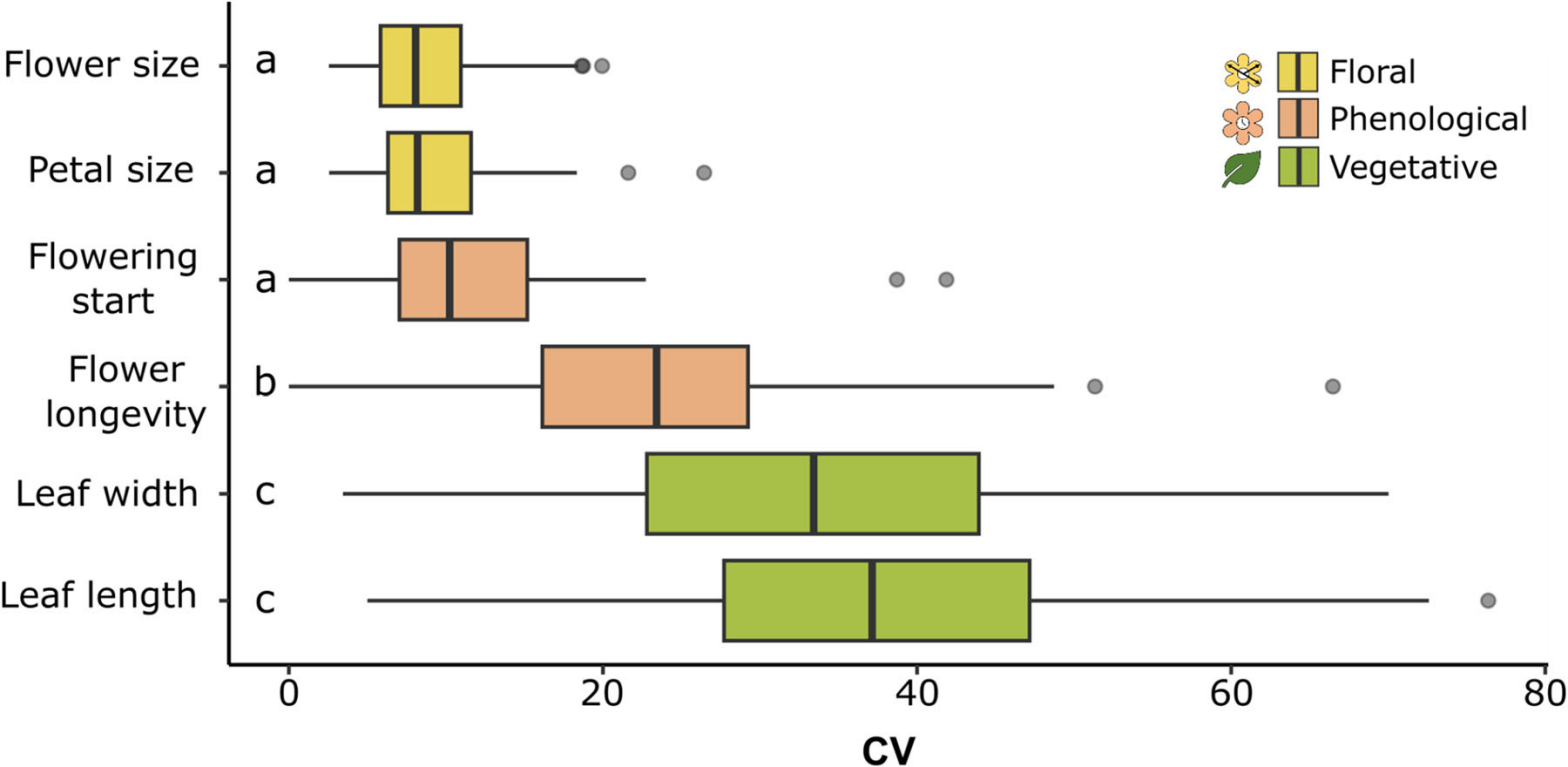
Intraindividual variation (measured as the coefficient of variation [CV]) of flower size, petal size, flowering start, flower longevity, leaf width, and leaf length at the intraramet level in *Galium odoratum* and derived from the complete data set (CV_ALL_). The trait types (floral, phenology, and vegetative) are indicated by color and icon. Different letters indicate a significant difference based on a post hoc Tukey test among the traits.

Intraindividual variation, assessed as CV_ALL_ within ramets across all traits, differed significantly among traits (Table 1; Figure 1). Flower size (mean CV ± SE: 8.8 ± 4.1), petal size (9.2 ± 4.1 SE), and flowering start (11.2 ± 6.3 SE) displayed the least intraindividual variation. Flower longevity (24.1 ± 10.4 SE) had an intermediate amount of intraindividual variation, and leaf width (33.4 ± 14.6 SE) and leaf length (37.9 ± 14.8 SE) had the most intraindividual variation among all measured traits (Figure 1; Appendices S7, S8).

**Table 1:** Results of the mixed‐effects model of intraindividual variation (measured as the coefficient of variation (CV) calculated within each trait in the complete data set [CV_ALL_]) in *Galium odoratum* at the intraramet level as response variable.

Response	Predictors	*χ* ^2^	df	*P*
CV	Trait	1165.22	5	**<0.001**
	Trait mean	67.91	1	**<0.001**
	Treatment	9.85	3	**0.020**
	Trait:Treatment	12.50	15	0.641

	**Variance**	**SD**
Region/Population/Genet^a^	0.008	0.089

Trait, mean trait value, the experimental treatments and the interaction between trait and treatment were included as fixed factors. Genet nested within population and region was included as random factor. Variance and standard deviation (SD) are provided for the random factor. Significant *P*‐values are bolded.

^a^ Genet:Population:Region sample size (*N*) = 65, Population:Region *N* = 21, Region = 3.

### Treatment effects on intraindividual variation and trait means

**Figure 2:**
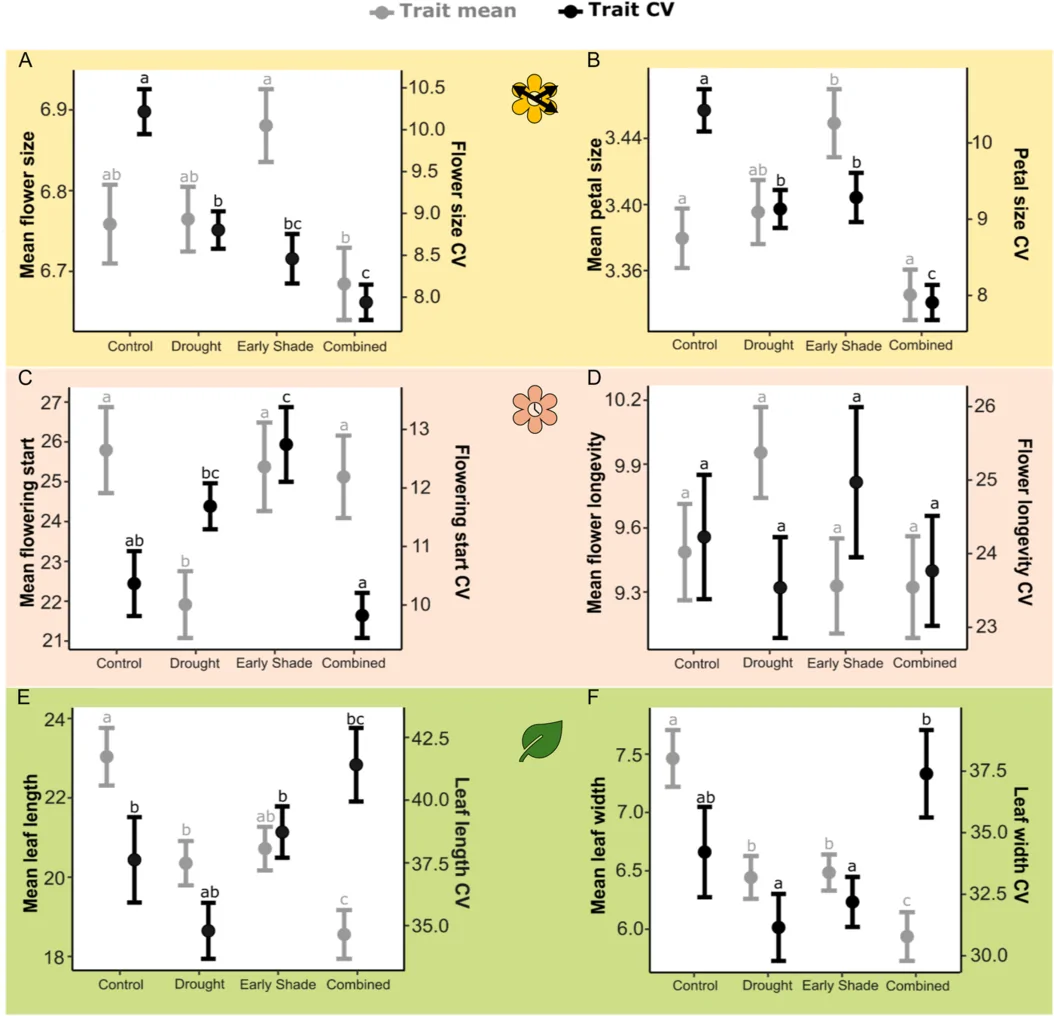
Treatment effects on the predicted intraindividual variation (measured as the coefficient of variation [CV]) and predicted means of (A) flower size, (B) petal size, (C) flowering start, (D) flower longevity, (E) leaf length, and (F) leaf width at the intraramet level in *Galium odoratum*. The trait types (floral, phenology, and vegetative) are indicated by color and icon.

We found a significant effect of the treatments on both the overall intraindividual variation across all traits (CV_ALL_; Table 1) and on some trait‐specific intraindividual variation (Table 2; see Appendix S9 and Appendix S10 for the models on trait means and trait SDs, respectively). While the overall LMMs showed limited significance for the treatment effects (Table 2), the post hoc Tukey tests revealed treatment‐specific significant differences (Figure 2; Appendix S11). More specifically, the intraindividual variation of flower and petal size was significantly higher under control conditions (Figure 2A, B; Appendix S11), significantly lower under combined treatment conditions, and somewhat intermediate under drought and early shading. In comparison, mean floral trait responses were mostly of equal size across treatments, except for under early shading, where mean flower and petal size were significantly higher (Figure 2A, B). Furthermore, flowering start had increased intraindividual variation in the drought treatment and the early shading treatment (Figure 2C). In comparison, mean flowering start was similar across treatments, except for under the drought treatment, where individuals flowered significantly earlier (Figure 2C). No trends in intraindividual variation nor the mean of flower longevity among treatments were observed (Figure 2D). Finally, intraindividual variation of leaf length and leaf width increased significantly under combined treatments (Figure 2E, F), while vegetative trait means were also found to decrease significantly under the drought and the early‐shade treatments compared to the control, with an even further decrease in the combined treatment (Figure 2E, F).

**Table 2:** Effects of the applied treatments on trait‐specific variation at the intraramet level. Intraindividual variation (measured as the coefficient of variation [CV] and calculated for each trait at the intraramet level) in *Galium odoratum* was used as response variables.

Response variable	Predictor	Estimate	*χ* ^2^	df	*P*
Flowering start CV	Trait mean	0.000	0.378	1	0.539
	Shade	0.012	0.178	1	0.673
	Drought	**0.001**	**1.249**	1	**0.264**
	Shade:Drought	–0.017	1.570	1	0.210
Flower longevity CV	Trait mean	–0.019	18.009	1	**<0.001**
	Shade	0.003	0.038	1	0.845
	Drought	0.001	0.354	1	0.552
	Shade:Drought	–0.013	0.184	1	0.668
Flower size CV	Trait mean	–0.014	9.102	1	**0.003**
	Shade	–0.016	3.115	1	0.078
	Drought	–0.015	2.615	1	0.106
	Shade:Drought	0.007	0.204	1	0.652
Petal size CV	Trait mean	–0.042	7.781	1	**0.005**
	Shade	–0.025	3.755	1	0.053
	Drought	–0.026	4.341	1	**0.040**
	Shade:Drought	0.006	0.056	1	0.807
Leaf length CV	Trait mean	–0.012	11.433	1	**<0.001**
	Shade	–0.009	0.017	1	0.896
	Drought	–0.050	1.450	1	0.229
	Shade:Drought	0.026	0.186	1	0.666
Leaf width CV	Trait mean	–0.036	14.633	1	**<0.001**
	Shade	–0.054	0.098	1	0.754
	Drought	–0.075	1.193	1	0.275
	Shade:Drought	0.088	2.353	1	0.125

Trait mean, treatment, and their interaction were included as fixed factors and covariates. Genet nested within population and region was included as random factor. Significant *P*‐values are bolded.

### Relationships between intraindividual variation and trait means

**Figure 3:**
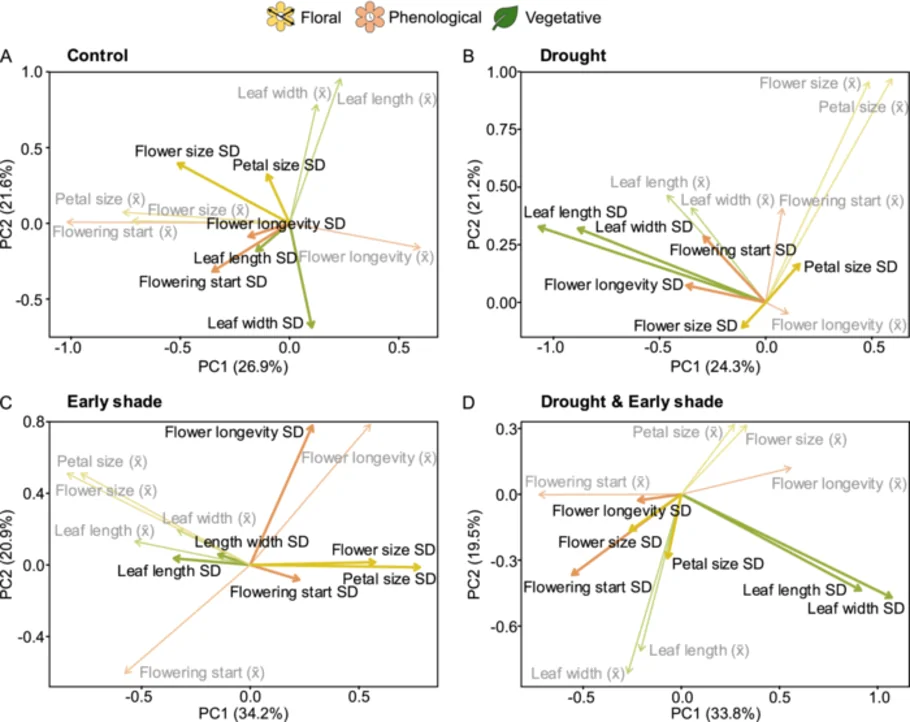
PCA of mean trait responses (gray text and lighter‐colored arrows) and intraindividual trait variation (measured as the SD); black text and darker‐colored arrows) for (A) the control treatment, (B) drought treatment, (C) early‐shade treatment, and (D) the combined drought and early‐shade treatment. In all cases, the first two axes (PC1 and PC2) are depicted, and the percentage of variation explained. Solid arrows represent the direction of the studied trait SDs and means. Trait types (floral, phenology and, vegetative) are indicated by icons, and their color matches the arrows.

According to the permutation‐based test, intraindividual trait variation in the functional trait space was nonrandom across all treatments. More specifically, in the control treatment, PC1 and PC2 were statistically significant (*P* = 0.004 and *P* < 0.0001, respectively) and therefore exceeded the null threshold. The PCA showed that under the control treatment (Figure 3A), intraindividual variation and trait means were determined almost equally by PC1 (26.9%) and PC2 (21.6%). Significant positive relationships were observed among the intraindividual variations within the same trait types (i.e., floral, phenological and vegetative trait types), whereas strong, negative correlations primarily occur among trait means (Figure 3A; Appendix S12). Furthermore, a significant negative correlation was found between intraindividual variation in flower size and mean flower longevity (Figure 3A; Appendix S12). In the drought treatment, PC1 and PC2 were statistically significant (*P* = 0.004 and *P* < 0.0001, respectively) and therefore exceeded the null threshold. Furthermore, intraindividual variation and trait means were defined almost equally by PC1 (24.3%) and PC2 (21.2%) (Figure 3B). More specifically, in the drought treatment, we primarily observed significant positive relationships of intraindividual variation in vegetative and phenological traits, whereas intraindividual variation in floral traits were significantly negatively correlated (Figure 3B; Appendix S12). In the early‐shading treatment, only PC1 was statistically significant (*P* < 0.0001), whereas PC2 did not exceed the null threshold. Furthermore, PC1 (34.2%) defined a greater part compared to PC2 (20.9%) (Figure 3C). Intraindividual variation primarily had significantly positive correlations within trait types, but among trait types, we could observe significantly negative relationships between intraindividual variation in floral traits and intraindividual variation in vegetative traits (Figure 3C; Appendix S12). Lastly, for the combined treatment, both PC1 (*P* < 0.0001) and PC2 (*P* < 0.0001) were significant and exceeded the null threshold. PC1 (33.8%) defined substantially more than PC2 (19.5%) (Figure 3D). Intraindividual variation was again positively correlated within trait types. However, significant negative correlations were observed for intraindividual variation in vegetative traits and mean flowering start (Figure 3D; Appendix S12).

## DISCUSSION

Here, we showed that intraindividual variation varied strongly among traits, with the least variation found in floral traits, intermediate amounts in phenological traits, and the most in vegetative traits, concurrent with patterns at the interindividual level (Schlichting and Levin, 1984; Frazee and Marquis, 1994; Villellas et al., 2021). Furthermore, we showed that intraindividual variation was induced by experimental drought, early shading, and/or their interaction, with the direction of the response depending on the trait. Lastly, we revealed distinct multidimensional functional trait spaces and environment‐induced dynamics in the relationships within and between the intraindividual variation trait mean of various traits. These findings suggest that intraindividual variation may play an important role for plants to adjust and thrive under global environmental change.

### Differences in intraindividual variation among plant traits

The measured traits differed in the extent of their intraindividual variation. We found the highest intraindividual variation in the vegetative traits, leaf length and leaf width, concurrent with previous findings (Herrera, 2009). Greater variation in leaf traits may allow individuals to thrive under variable light and drought conditions, simultaneously increasing light‐capturing ability and decreasing potential evapotranspiration (Møller et al., 2023). Floral traits, such as petal and flower size, had low levels of intraramet variation. Flower size has been reported to be under strong selection exerted by pollinators (Herrera, 2009; Parachnowitsch and Kessler, 2010), offering a potential explanation for the low trait variation within flowers. Small variation in floral traits has previously been shown to have important evolutionary consequences for pollinator interactions, albeit only on the inter‐ and intraspecific level (Lambrecht and Dawson, 2007). In contrast, increased flower size variation within an individual could accommodate more pollinator species (Gómez et al., 2020). Low intraindividual variation was also found for flowering start, whereas flower longevity was more variable. Timing and response to environmental cues in phenological traits such as flowering start are crucial to maintain fitness (Elzinga et al., 2007; Pau et al., 2011). Hence, the low intraindividual variation for flowering start in our common garden might be the result of strong selection for simultaneous flowering when pollinators are present and potential risks (such as late frosts) are low. Furthermore, many plant species are advancing their phenology (Johansson et al., 2013), and our observed low variation in flowering start might very well indicate that this pattern might be so strong that it applies to all flowers, ultimately lowering the variation. In contrast, the intermediate amount of intraindividual variation in flower longevity suggests that, while flowering start is under strong genetic constraints, individuals can vary the individual longevity of a flower in response to the environment. For instance, flowers may finish anthesis after successful pollination (Primack, 1985; Trunschke and Stöcklin, 2017).

### Treatment effects on intraindividual variation and trait means

We observed parallel treatment effects, but also contrasting in direction and magnitude, on intraindividual variation and trait means. For the vegetative traits, the drought treatment led to reduced variation. One explanation could be that genetic and developmental constraints on leaf growth and morphology become apparent under drought stress (Farooq et al., 2009, 2012), which may limit the capacity for high intraindividual variation in leaf traits. Leaves that develop with more sun exposure may remain relatively small and thick to reduce the risk of water stress (McDowell et al., 2008; Nardini et al., 2014), while leaves that develop in more shade may have larger leaf area to maximize light capture (Weijschedé et al., 2006). High variation in vegetative traits was observed with low trait means in the combined treatment. As opposed to the drought treatment, the increased variation in vegetative traits under the combined treatment might be a result of diversification of resource allocation.

For floral traits, intraindividual variation was highest in the benign control conditions and decreased with progressively stressful conditions. In the control conditions, resources allocated to floral traits might allow for great variation in flower and petal size, accommodating a greater range of pollinators, in the same way that a greater range of fruit sizes attracts more‐varied dispersers (Sobral, 2023). Under early shading, trait means of floral traits slightly increased compared to the control conditions, enhancing the floral display. Enhanced floral displays may help attract pollinators, which are often scarce in the forest understorey, while competition for pollinators among plants is high. However, under the combined treatment, both intraindividual variation and floral trait means were at their lowest. These differing responses to the various treatments could indicate that intraindividual variation in flower and petal size and large means in these traits might not be a priority for the individual under severe resource constraints, although this interpretation needs further evidence.

Regarding phenological traits, we mainly found low means for flowering start and thus earlier flowering in individuals exposed to drought, which could indicate a drought escape attempt (Shavrukov et al., 2017). However, flowering start showed a more enigmatic pattern in its intraindividual variation, with low variation in the two extremes—the control and the combined treatment—and with high variation under drought and early‐shading separately, which we cannot explain. Finally, a distinct pattern in variation of flower longevity or in its average was not found, indicating that longevity might be a constrained trait with little to no plasticity available. An alternative explanation could be that the trait variation among individuals within treatments is as high as the trait variation observed among treatments due to some unknown, potentially physiological factor(s).

Our results showed that intraindividual variation in most of the measured traits responded to the simulated future climatic conditions and is therefore inducible, highlighting the potential importance of intraindividual variation for rapid adjustment to the environment and for organismal fitness.

### Relationships between intraindividual variation and trait means

Our PCA results showed that, in a multidimensional trait space, the relationships within and between intraindividual variation and the corresponding trait means were altered depending on the treatments, indicating that these relationships are dynamic and can be environmentally induced.

All types of relationships (i.e., positive, negative, and neutral) were found but in different proportions depending on the treatment. We consistently saw patterns of positive relationships of intraindividual variation within trait types (floral, phenological, and vegetative) across treatments. These positive relationships could be indications of phenotypic integration (Pigliucci, 2003; Matesanz et al., 2021), reflected by trait covariance where multiple traits respond in a coordinated way due to a shared underlying genetic, developmental, or environmental drivers. Furthermore, some negative relationships between intraindividual variation and trait means also occurred among trait types, mainly under treatments mimicking environmental stress. Here, there might be a trade‐off between the variation of one trait and the mean trait response of another, reflecting a potential genetic constraint or ecological pressure (Southwood, 1988). Lastly, the majority of the trait relationship were negligible, which could reflect independence where intraindividual variation can be considered as an individual property in itself or perhaps be a result of benign treatment conditions.

Overall, we showed changes in the patterns of relationships across treatments, indicating dynamic relationships between intraindividual variation and trait means driven by environmental conditions. These dynamic relationships signal that our simulated forest conditions can alter the plants’ responses, implying that changes in intraindividual variation and in trait means are key, coordinated components in plant strategies. Based on our findings, we can speculate that, while too little intraindividual trait variation could put an individual at a disadvantage when faced with environmental change, too much variation could block the functions of each trait type and local adaptations by the individuals (Kawecki and Ebert, 2004) from solidifying.

### Future directions

Our study lacked a direct measure of fitness, which is crucial for determining the adaptive significance of the observed patterns. The inclusion of fitness metrics in future experiments will enable us to assess whether the observed patterns are an adaptive function of intraindividual variation on its own or merely a passive response. Furthermore, direct measurements of the quantity and diversity of floral visitors will help us understand dependent ecosystem functions. Additionally, despite our efforts to reduce any potential positional effects (e.g., by only including the leaf measurements from the top whorl), we did not account for ontogenetic patterns, which might influence our results. For example, flower size may decrease as the inflorescence grows, which would inevitably result in greater intraindividual variation. Future work could explicitly account for ontogenetic and positional effects by sampling organs at standardized developmental stages. Finally, long‐term studies will provide a more comprehensive understanding of how the observed patterns develop and persist over time, which can help to determine whether the responses are consistent with responses to the treatments and stable indicators of adaptive strategies (i.e., whether they fluctuate with environmental change).

## CONCLUSIONS

The magnitude of intraindividual variation differed greatly among plant traits in our study species. The lowest values were found in floral traits, followed by phenological traits, with the highest in vegetative traits. Furthermore, intraindividual variation was environmentally inducible because it responded to the drought and shade treatments, indicating the value of intraindividual trait variation for plants to deal with current changes in forest and other environments. Additionally, our study showed that the trait variation originating from distinct plant traits and trait types are dominant dimensions of the trait spectrum alongside trait averages. Lastly, the ecological meaning behind the observed dynamics remains unknown but could potentially indicate that trait variation shifts with environmental conditions to maintain plant functionality in different contexts. Understanding the dynamics of intraindividual variation is crucial for predicting functional strategies that plants might utilize in response to future climatic changes.

## AUTHOR CONTRIBUTIONS

C.M., M.M.S., P.D.F., and J.F.S. designed the study. C.M. did the field sampling and J.H. measured traits in the common garden experiment. C.M., M.M.S., and M.S. analyzed the data. C.M. wrote the first draft of the manuscript with all coauthors contributing to revisions.

## CONFLICT OF INTEREST STATEMENT

The authors declare no conflicts of interest.

## Supporting information


**Appendix S1:** Overview of distribution of individuals included in the study design for floral traits, treatments, number of genets, and number of sampled populations in the three regions.

**Appendix S2:** Overview of distribution of individuals included in the study design for vegetative traits, treatments, number of genets, and number of sampled populations that were sampled in the three regions.

**Appendix S3:** Contributions of trait means and intraindividual trait variation (measured as standard deviation) for principal components 1–4 for the control treatment.

**Appendix S4:** Contributions of trait means and intraindividual trait variation (measured as standard deviation) for principal components 1–4 for the drought treatment.

**Appendix S5:** Contributions of the trait means and intraindividual trait variation (measured as standard deviation; SD) for principal components 1–4 for the early‐shading treatment.

**Appendix S6:** Contributions of trait means and intraindividual trait variation (measured as standard deviation) for principal components 1–4 for the combined drought and early‐shade treatment.

**Appendix S7:** Pairwise comparisons of intraindividual variation (measured as the coefficient of variation) among traits using a post hoc Tukey test.

**Appendix S8:** Pairwise comparisons of intraindividual variation (measured as the coefficient of variation; CV) among traits using a post hoc Tukey test.

**Appendix S9:** Effects of the applied treatments on trait‐specific means at the intraramet level.

**Appendix S10:** Effects of the applied treatments on trait‐specific variation at the intraramet level.

**Appendix S11:** Experimental treatment differences for intraindividual variation for each trait and trait mean.

**Appendix S12:** Correlation matrix showing Pearson's pairwise correlation coefficients for the measured trait means and intraindividual trait variation for (a) control; (b) drought; (c) early shade; and (d) combined drought and early shading treatment.

## Data Availability

This work is based on data elaborated by the HerbAdapt project of the Biodiversity Exploratories program (DFG Priority Program 1374). The data sets are publicly available from the Biodiversity Exploratories Information System (10.17616/R32P9Q) at https://www.bexis.uni-jena.de/ddm/publicsearch/ under ID 31766 and 31762.
