# Supplementary material for: Environmentally induced dynamics between intraindividual variation and trait means across vegetative, phenological, and floral traits
Source: Am J Bot. 2026 Aug 7;113(8):e70244. doi: 10.1002/ajb2.70244 (PMC13496112; doi:10.1002/ajb2.70244)
Supplement: Supplementary file 1 [file AJB2-113-e70244-s001.docx]

**Appendix S8.** Pairwise comparisons of intraindividual variation (measured as the CV) among traits using a post hoc Tukey test. Significant *P*-values are bolded. Estimates, standard errors and *t*-values across all combinations of traits and treatments are for the model correspond to Table 2.

| **Comparison** | **Estimate** | **SE** | ***t*-ratio** | ***P*** |
| --- | --- | --- | --- | --- |
| Flower longevity vs flower size | 0.189 | 0.013 | 14.952 | **<0.0001** |
| Flower longevity vs flowering start | 0.156 | 0.013 | 12.365 | **<0.0001** |
| Flower longevity vs leaf length | -0.122 | 0.014 | -8.927 | **<0.0001** |
| Flower longevity vs leaf width | -0.083 | 0.014 | -6.025 | **<0.0001** |
| Flower longevity vs petal size | 0.183 | 0.013 | 14.458 | **<0.0001** |
| Flower size vs flowering start | -0.033 | 0.013 | -2.586 | 0.102 |
| Flower size vs leaf length | -0.312 | 0.014 | -22.719 | **<0.0001** |
| Flower size vs leaf width | -0.272 | 0.014 | -19.818 | **<0.0001** |
| Flower size vs petal size | -0.006 | 0.013 | -0.494 | 0.996 |
| Flowering start vs leaf length | -0.279 | 0.014 | -20.334 | **<0.0001** |
| Flowering start vs leaf width | -0.239 | 0.014 | -17.432 | **<0.0001** |
| Flowering start vs petal size | 0.026 | 0.013 | 2.093 | 0.293 |
| Leaf length vs leaf width | 0.040 | 0.014 | 2.756 | 0.066 |
| Leaf length vs petal size | 0.305 | 0.014 | 22.264 | **<0.0001** |
| Leaf width vs petal size | 0.266 | 0.014 | 19.363 | **<0.0001** |
