# Supplementary material for: Environmentally induced dynamics between intraindividual variation and trait means across vegetative, phenological, and floral traits
Source: Am J Bot. 2026 Aug 7;113(8):e70244. doi: 10.1002/ajb2.70244 (PMC13496112; doi:10.1002/ajb2.70244)
Supplement: Supplementary file 2 [file AJB2-113-e70244-s002.docx]

**Appendix S9.** Effects of the applied treatments on trait-specific means at the intraramet level. Trait mean in *Galium odoratum* at the intraramet level calculated for each trait was used as the response variable. Intraindividual variation (measured as CV), treatment, and their interaction were included as fixed factors and covariates. Genet nested within population and region was included as random factor. Significant *P*-values are bolded.

| **Response variable** | **Predictor** | ***χ*^2^** | **df** | ***P*** |
| --- | --- | --- | --- | --- |
| Mean leaf length | Trait CV | 11.729 | 1 | **<0.001** |
|  | Shade | 3.510 | 1 | 0.061 |
|  | Drought | 11.708 | 1 | **<0.001** |
|  | Shade:Drought | 0.613 | 1 | 0.434 |
| Mean leaf width | Trait CV | 14.340 | 1 | **<0.001** |
|  | Shade | 3.890 | 1 | **0.049** |
|  | Drought | 9.659 | 1 | **0.002** |
|  | Shade:Drought | 0.043 | 1 | 0.836 |
| Mean flowering start | Trait CV | 0.897 | 1 | 0.345 |
|  | Shade | 6.073 | 1 | **0.014** |
|  | Drought | 1.128 | 1 | 0.288 |
|  | Shade:Drought | 1.246 | 1 | 0.264 |
| Mean flower longevity | Trait CV | 22.541 | 1 | **<0.001** |
|  | Shade | 1.200 | 1 | 0.273 |
|  | Drought | 0.446 | 1 | 0.504 |
|  | Shade:Drought | 0.280 | 1 | 0.597 |
| Mean flower size | Trait CV | 9.647 | 1 | **0.002** |
|  | Shade | 0.088 | 1 | 0.767 |
|  | Drought | 1.046 | 1 | 0.306 |
|  | Shade:Drought | 0.257 | 1 | 0.612 |
| Mean petal size | Trait CV | 7.386 | 1 | **0.007** |
|  | Shade | 0.082 | 1 | 0.775 |
|  | Drought | 1.028 | 1 | 0.311 |
|  | Shade:Drought | 0.712 | 1 | 0.399 |
