# Supplementary material for: Environmentally induced dynamics between intraindividual variation and trait means across vegetative, phenological, and floral traits
Source: Am J Bot. 2026 Aug 7;113(8):e70244. doi: 10.1002/ajb2.70244 (PMC13496112; doi:10.1002/ajb2.70244)
Supplement: Supplementary file 3 [file AJB2-113-e70244-s003.docx]

**Appendix S12.** Correlation matrix showing Pearson’s pairwise correlation coefficients for the measured trait means (*X̄*) and intraindividual trait variation (measured as standard deviation; SD) for (A) control; (B) drought; (C) early shade; and (D) combined drought and early-shade treatments. Significant correlations are outlined by a dashed box.
