# Supplementary material for: Environmentally induced dynamics between intraindividual variation and trait means across vegetative, phenological, and floral traits
Source: Am J Bot. 2026 Aug 7;113(8):e70244. doi: 10.1002/ajb2.70244 (PMC13496112; doi:10.1002/ajb2.70244)
Supplement: Supplementary file 4 [file AJB2-113-e70244-s004.docx]

**Appendix S1.** Overview of the distribution of the individuals (ramets) included in the study design for the floral traits (flowering start, flower longevity, flower size, and petal size), treatments, number of genets they originated from and number of populations that were sampled in each region.

| **Region** | **No. of populations** | **No. of genets** | **Treatment** | **No. of ramets** | **Total ramets measured** |
| --- | --- | --- | --- | --- | --- |
| **Schwäbian Alb (ALB)** | 8 | 21 | Control | 10 | 168 |
|  |  |  | Early Shading | 7 | 93 |
|  |  |  | Drought | 14 | 202 |
|  |  |  | Early Shading + Drought | 5 | 75 |
| **Hanich-Dün (HAI)** | 8 | 26 | Control | 11 | 147 |
|  |  |  | Early Shading | 8 | 113 |
|  |  |  | Drought | 10 | 132 |
|  |  |  | Early Shading + Drought | 16 | 234 |
| **Schorfheide-Chorin (SCH)** | 5 | 17 | Control | 5 | 74 |
|  |  |  | Early Shading | 9 | 183 |
|  |  |  | Drought | 13 | 134 |
|  |  |  | Early Shading + Drought | 5 | 66 |

| **Total** | 21 | 65 | Control | 26 | 389 |
| --- | --- | --- | --- | --- | --- |
|  |  |  | Early Shading | 24 | 389 |
|  |  |  | Drought | 37 | 468 |
|  |  |  | Early Shading + Drought | 26 | 375 |
|  |  |  | **Total** | **113** | **1621** |
