# Supplementary material for: Environmentally induced dynamics between intraindividual variation and trait means across vegetative, phenological, and floral traits
Source: Am J Bot. 2026 Aug 7;113(8):e70244. doi: 10.1002/ajb2.70244 (PMC13496112; doi:10.1002/ajb2.70244)
Supplement: Supplementary file 7 [file AJB2-113-e70244-s007.docx]

**Appendix S7.** Pairwise comparisons of intraindividual variation (measured as the CV) among traits using a post hoc Tukey test. Estimates, standard errors and t-value for the model corresponding to Table 1.

| **Fixed effects** | **Estimate** | **SE** | ***t*** |
| --- | --- | --- | --- |
| (Intercept) | 0.464 | 0.018 | 25.187 |
| Trait ‒ Flower size | -0.198 | 0.025 | -8.012 |
| Trait ‒ Flowering start | -0.164 | 0.025 | -6.630 |
| Trait ‒ Leaf length | 0.143 | 0.027 | 5.276 |
| Trait ‒ Leaf width | 0.106 | 0.027 | 3.899 |
| Trait ‒ Petal size | -0.196 | 0.025 | -7.931 |
| Trait mean | -0.032 | 0.004 | -8.241 |
| Treatment ‒ Control | 0.019 | 0.025 | 0.768 |
| Treatment ‒ Drought | 0.012 | 0.024 | 0.486 |
| Treatment ‒ Early shade | 0.026 | 0.025 | 1.045 |
| Treatment ‒ Flower size × Treatment ‒ Control | 0.027 | 0.035 | 0.769 |
| Treatment ‒ Flowering start × Treatment ‒ Control | -0.003 | 0.035 | -0.089 |
| Treatment ‒ Leaf length × Treatment ‒ Control | -0.006 | 0.039 | -0.149 |
| Treatment ‒ Leaf width × Treatment ‒ Control | 0.003 | 0.039 | 0.069 |
| Treatment ‒ Petal size × Treatment ‒ Control | 0.029 | 0.035 | 0.834 |
| Treatment ‒ Flower size × Treatment ‒ Drought | 0.011 | 0.033 | 0.334 |
| Treatment ‒ Flowering start × Treatment ‒ Drought | 0.009 | 0.033 | 0.257 |
| Treatment ‒ Leaf length × Treatment ‒ Drought | -0.055 | 0.036 | -1.548 |
| Treatment ‒ Leaf width × Treatment ‒ Drought | -0.049 | 0.036 | -1.361 |
| Treatment ‒ Petal size × Treatment ‒ Drought | 0.014 | 0.033 | 0.411 |
| Treatment ‒ Flower size × Treatment ‒ Early shade | -0.002 | 0.035 | -0.058 |
| Treatment ‒ Flowering start × Treatment ‒ Early shade | 0.024 | 0.035 | 0.698 |
| Treatment ‒ Leaf length × Treatment ‒ Early shade | -0.010 | 0.037 | -0.269 |
| Treatment ‒ Leaf width × Treatment ‒ Early shade | -0.033 | 0.037 | -0.900 |
| Treatment ‒ Petal size × Treatment ‒ Early shade | 0.010 | 0.035 | 0.294 |
