# Supplementary material for: Environmentally induced dynamics between intraindividual variation and trait means across vegetative, phenological, and floral traits
Source: Am J Bot. 2026 Aug 7;113(8):e70244. doi: 10.1002/ajb2.70244 (PMC13496112; doi:10.1002/ajb2.70244)
Supplement: Supplementary file 8 [file AJB2-113-e70244-s008.docx]

**Appendix S10.** Effects of the applied treatment on trait-specific variation at the intraramet level. Intraindividual variation (measured as SD and calculated at the intraramet level for each trait) in *Galium odoratum* was used as the response variable. Trait mean, treatment, and their interaction were included as fixed factors and covariates. Genet nested within population and region was included as random factor. Significant *P*-values are bolded.

| **Response variable** | **Predictor** | ***χ*^2^** | **df** | ***P*** |
| --- | --- | --- | --- | --- |
| Flowering start SD | Trait mean | 3.857 | 1 | **0.050** |
|  | Shade | 0.150 | 1 | 0.699 |
|  | Drought | 1.289 | 1 | 0.256 |
|  | Shade:Drought | 1.446 | 1 | 0.229 |
| Flower longevity SD | Trait mean | 1.304 | 1 | 0.253 |
|  | Shade | 0.000 | 1 | 0.991 |
|  | Drought | 0.181 | 1 | 0.671 |
|  | Shade:Drought | 0.250 | 1 | 0.617 |
| Flower size SD | Trait mean | 0.028 | 1 | 0.867 |
|  | Shade | 3.321 | 1 | 0.068 |
|  | Drought | 2.857 | 1 | 0.091 |
|  | Shade:Drought | 0.335 | 1 | 0.563 |
| Petal size SD | Trait mean | 0.084 | 1 | 0.772 |
|  | Shade | 3.849 | 1 | **0.050** |
|  | Drought | 3.850 | 1 | **0.050** |
|  | Shade:Drought | 0.116 | 1 | 0.733 |
| Leaf length SD | Trait mean | 3.012 | 1 | 0.083 |
|  | Shade | 0.039 | 1 | 0.844 |
|  | Drought | 1.488 | 1 | 0.223 |
|  | Shade:Drought | 0.016 | 1 | 0.901 |
| Leaf width SD | Trait mean | 0.145 | 1 | 0.703 |
|  | Shade | 0.252 | 1 | 0.616 |
|  | Drought | 2.599 | 1 | 0.107 |
|  | Shade:Drought | 1.144 | 1 | 0.285 |
