# Supplementary material for: Environmentally induced dynamics between intraindividual variation and trait means across vegetative, phenological, and floral traits
Source: Am J Bot. 2026 Aug 7;113(8):e70244. doi: 10.1002/ajb2.70244 (PMC13496112; doi:10.1002/ajb2.70244)
Supplement: Supplementary file 9 [file AJB2-113-e70244-s009.docx]

**Appendix S6.** Contributions of the trait means and intraindividual trait variation (measured as standard deviation; SD) for (A) principal component 1 (PC1); (B) principal component 2 (PC2); (C) principal component 3 (PC3); and (D) principal component 4 (PC4) for the combined drought and early shading treatment.
