# Supplementary material for: Environmentally induced dynamics between intraindividual variation and trait means across vegetative, phenological, and floral traits
Source: Am J Bot. 2026 Aug 7;113(8):e70244. doi: 10.1002/ajb2.70244 (PMC13496112; doi:10.1002/ajb2.70244)
Supplement: Supplementary file 10 [file AJB2-113-e70244-s010.docx]

**Appendix S2.** Overview of the distribution of the individuals included in the study design for vegetative traits (leaf length and leaf width), treatments, the number of genets they originated from and the number of populations that had successfully been sampled in each of the three regions: Schwäbian Alb, Hainich-Dün, and Schorfheide-Chorin.

| **Region** | **No. of populations** | **No. of genets** | **Treatment** | **No. ramets measured** |
| --- | --- | --- | --- | --- |
| **Schwäbian Alb (ALB)** | 4 | 10 | Control | 14 |
|  |  |  | Early Shading | 15 |
|  |  |  | Drought | 35 |
|  |  |  | Early Shading + Drought | 4 |
| **Hanich-Dün (HAI)** | 6 | 24 | Control | 48 |
|  |  |  | Early Shading | 40 |
|  |  |  | Drought | 50 |
|  |  |  | Early Shading + Drought | 70 |
| **Schorfheide-Chorin (SCH)** | 5 | 17 | Control | 25 |
|  |  |  | Early Shading | 60 |
|  |  |  | Drought | 44 |
|  |  |  | Early Shading + Drought | 20 |

| **Total** | 15 | 51 | Control | 87 |
| --- | --- | --- | --- | --- |
|  |  |  | Early Shading | 115 |
|  |  |  | Drought | 129 |
|  |  |  | Early Shading + Drought | 94 |
|  |  |  | **Total** | **425** |
