# Supplementary material for: Environmentally induced dynamics between intraindividual variation and trait means across vegetative, phenological, and floral traits
Source: Am J Bot. 2026 Aug 7;113(8):e70244. doi: 10.1002/ajb2.70244 (PMC13496112; doi:10.1002/ajb2.70244)
Supplement: Supplementary file 11 [file AJB2-113-e70244-s011.docx]

**Appendix S11.** Differences among treatments for intraindividual variation (measured as the coefficient of variation [CV]) for each trait and trait mean. Results are from post hoc Tukey tests based on the models from Table 3. Estimates, standard errors (SE), degrees of freedom (df) according to the Kenward–Roger method, *t*-ratio, and *P*-values. Significant P-values are bolded.

| **Leaf length CV** | | | | | |
| --- | --- | --- | --- | --- | --- |
| Comparison | Estimate | SE | df | *t*-ratio | *P* |
| Control ‒ Early shade | -0.006 | 0.0168 | 66.8 | -0.328 | 0.988 |
| Control ‒ Drought | 0.023 | 0.0163 | 73.3 | 1.332 | 0.546 |
| Control ‒ Combined | -0.029 | 0.0174 | 65.5 | -1.656 | 0.355 |
| Early shade ‒ Drought | 0.027 | 0.0151 | 70.5 | 1.796 | 0.284 |
| Early shade ‒ Combined | -0.023 | 0.0170 | 74.3 | -1.370 | 0.522 |
| Drought ‒ Combined | -0.051 | 0.0164 | 70.3 | -3.072 | **0.016** |
| **Leaf length mean** | | | | | |
| Comparison | Estimate | SE | df | *t*-ratio | *P* |
| Control ‒ Early shade | 1.08 | 0.516 | 66.9 | 2.101 | 0.164 |
| Control ‒ Drought | 2.22 | 0.499 | 73.3 | 4.459 | **<0.001** |
| Control ‒ Combined | 4.90 | 0.534 | 65.5 | 9.183 | **<0.001** |
| Early shade ‒ Drought | 1.14 | 0.464 | 70.4 | 2.458 | 0.076 |
| Early shade ‒ Combined | 3.82 | 0.522 | 74.2 | 7.327 | **<0.001** |
| Drought ‒ Combined | 2.68 | 0.504 | 70.1 | 5.322 | **<0.001** |
| **Leaf width CV** | | | | | |
| Comparison | Estimate | SE | df | *t*-ratio | *P* |
| Control ‒ Early shade | 0.042 | 0.0182 | 49.9 | 2.306 | 0.110 |
| Control ‒ Drought | 0.043 | 0.0181 | 56.9 | 2.379 | 0.093 |
| Control ‒ Combined | -0.020 | 0.0188 | 51.0 | -1.071 | 0.709 |
| Early shade ‒ Drought | 0.001 | 0.0170 | 58.3 | 0.071 | 0.999 |
| Early shade ‒ Combined | -0.062 | 0.0193 | 60.9 | -3.215 | **0.011** |
| Drought ‒ Combined | -0.063 | 0.0184 | 58.9 | -3.443 | **0.006** |
| **Leaf width mean** | | | | | |
| Comparison | Estimate | SE | df | *t*-ratio | *P* |
| Control ‒ Early shade | 0.641 | 0.206 | 62.8 | 3.107 | **0.015** |
| Control ‒ Drought | 0.926 | 0.201 | 70.1 | 4.611 | **<0.001** |
| Control ‒ Combined | 1.567 | 0.213 | 62.2 | 7.343 | **<0.001** |
| Early shade ‒ Drought | 0.285 | 0.187 | 68.3 | 1.525 | 0.428 |
| Early shade ‒ Combined | 0.927 | 0.211 | 72.2 | 4.398 | **<0.001** |
| Drought ‒ Combined | 0.642 | 0.203 | 68.0 | 3.168 | **0.012** |
| **Flower size CV** | | | | | |
| Comparison | Estimate | SE | df | *t*-ratio | *P* |
| Control ‒ Early shade | 0.019 | 0.0032 | 77.3 | 5.911 | **<0.001** |
| Control ‒ Drought | 0.015 | 0.0031 | 82.9 | 4.748 | **<0.001** |
| Control ‒ Combined | 0.024 | 0.0032 | 74.5 | 7.455 | **<0.001** |
| Early shade ‒ Drought | -0.004 | 0.0030 | 82.7 | -1.447 | 0.474 |
| Early shade ‒ Combined | 0.005 | 0.0033 | 82.5 | 1.554 | 0.410 |
| Drought ‒ Combined | 0.010 | 0.0032 | 83.1 | 2.964 | **0.020** |
| **Flower size mean** | | | | | |
| Comparison | Estimate | SE | df | *t*-ratio | *P* |
| Control ‒ Early shade | -0.116 | 0.063 | 81.9 | -1.833 | 0.266 |
| Control ‒ Drought | -0.005 | 0.060 | 88.7 | -0.082 | 0.999 |
| Control ‒ Combined | 0.062 | 0.064 | 79.8 | 0.972 | 0.766 |
| Early shade ‒ Drought | 0.111 | 0.060 | 86.4 | 1.871 | 0.248 |
| Early shade ‒ Combined | 0.178 | 0.066 | 83.4 | 2.713 | **0.040** |
| Drought ‒ Combined | 0.067 | 0.063 | 84.5 | 1.061 | 0.714 |
| **Petal size CV** | | | | | |
| Comparison | Estimate | SE | df | *t*-ratio | *P* |
| Control ‒ Early shade | 0.0165 | 0.0028 | 75.1 | 5.971 | **<0.001** |
| Control ‒ Drought | 0.0151 | 0.0027 | 80.0 | 5.656 | **<0.001** |
| Control ‒ Combined | 0.0274 | 0.0028 | 72.0 | 9.855 | **<0.001** |
| Early shade ‒ Drought | -0.0014 | 0.0026 | 80.6 | -0.540 | 0.949 |
| Early shade ‒ Combined | 0.0109 | 0.0029 | 81.6 | 3.835 | **0.001** |
| Drought ‒ Combined | 0.0123 | 0.0028 | 81.3 | 4.464 | **<0.001** |
| **Petal size mean** | | | | | |
| Comparison | Estimate | SE | df | *t*-ratio | *P* |
| Control ‒ Early shade | -0.081 | 0.027 | 88.9 | -3.035 | **0.016** |
| Control ‒ Drought | -0.021 | 0.025 | 93.4 | -0.840 | 0.835 |
| Control ‒ Combined | 0.030 | 0.027 | 86.5 | 1.119 | 0.679 |
| Early shade ‒ Drought | 0.060 | 0.025 | 90.9 | 2.403 | 0.084 |
| Early shade ‒ Combined | 0.111 | 0.027 | 93.2 | 4.091 | **0.001** |
| Drought ‒ Combined | 0.051 | 0.026 | 93.8 | 1.961 | 0.210 |
| **Flowering start CV** | | | | | |
| Comparison | Estimate | SE | df | *t*-ratio | *P* |
| Control ‒ Early shade | -0.017 | 0.0053 | 63.0 | -3.236 | **0.010** |
| Control ‒ Drought | -0.009 | 0.0052 | 67.9 | -1.659 | 0.353 |
| Control ‒ Combined | 0.012 | 0.0053 | 61.3 | 2.303 | 0.109 |
| Early shade ‒ Drought | 0.009 | 0.0051 | 69.1 | 1.688 | 0.338 |
| Early shade ‒ Combined | 0.029 | 0.0056 | 68.5 | 5.272 | **<0.001** |
| Drought ‒ Combined | 0.021 | 0.0054 | 70.2 | 3.863 | **0.001** |
| **Flowering start mean** | | | | | |
| Comparison | Estimate | SE | df | *t*-ratio | *P* |
| Control ‒ Early shade | -1.162 | 0.749 | 52.5 | -1.552 | 0.414 |
| Control ‒ Drought | 3.068 | 0.738 | 55.5 | 4.158 | **0.001** |
| Control ‒ Combined | -1.256 | 0.743 | 52.1 | -1.692 | 0.338 |
| Early shade ‒ Drought | 4.230 | 0.735 | 56.0 | 5.757 | **<0.001** |
| Early shade ‒ Combined | -0.094 | 0.793 | 55.2 | -0.119 | 0.999 |
| Drought ‒ Combined | -4.324 | 0.766 | 56.9 | -5.647 | **<0.001** |
| **Flower longevity CV** | | | | | |
| Comparison | Estimate | SE | df | *t*-ratio | *P* |
| Control ‒ Early shade | -0.010 | 0.012 | 77.2 | -0.884 | 0.813 |
| Control ‒ Drought | 0.007 | 0.011 | 85.0 | 0.643 | 0.918 |
| Control ‒ Combined | 0.006 | 0.012 | 75.9 | 0.480 | 0.963 |
| Early shade ‒ Drought | 0.017 | 0.011 | 82.7 | 1.582 | 0.394 |
| Early shade ‒ Combined | 0.016 | 0.012 | 77.7 | 1.308 | 0.561 |
| Drought ‒ Combined | -0.001 | 0.011 | 80.9 | -0.129 | 0.999 |
| **Flower longevity mean** | | | | | |
| Comparison | Estimate | SE | df | *t*-ratio | *P* |
| Control ‒ Early shade | 0.342 | 0.291 | 72.2 | 1.175 | 0.644 |
| Control ‒ Drought | -0.377 | 0.278 | 79.3 | -1.358 | 0.529 |
| Control ‒ Combined | 0.242 | 0.291 | 70.9 | 0.830 | 0.840 |
| Early shade ‒ Drought | -0.720 | 0.277 | 78.3 | -2.599 | 0.053 |
| Early shade ‒ Combined | -0.101 | 0.306 | 74.5 | -0.329 | 0.988 |
| Drought ‒ Combined | 0.619 | 0.292 | 78.0 | 2.119 | 0.156 |
